# Utilizing Artificial Intelligence to Predict the Superplasticizer Demand of Self-Consolidating Concrete Incorporating Pumice, Slag, and Fly Ash Powders

**DOI:** 10.3390/ma14226792

**Published:** 2021-11-11

**Authors:** Jing Liu, Masoud Mohammadi, Yubao Zhan, Pengqiang Zheng, Maria Rashidi, Peyman Mehrabi

**Affiliations:** 1 Collage of Resources, Shandong University of Science and Technology, Taian 271019, China; bubushenglian1999@163.com (J.L.); pqzheng_1231@163.com (P.Z.); 2 Centre for Infrastructure Engineering, Western Sydney University, Penrith, NSW 2751, Australia; m.mohammadi@westernsydney.edu.au

**Keywords:** artificial neural network, prediction, superplasticizer demand, self-consolidating concrete, fresh properties, cementitious replacements

## Abstract

Self-consolidating concrete (SCC) is a well-known type of concrete, which has been employed in different structural applications due to providing desirable properties. Different studies have been performed to obtain a sustainable mix design and enhance the fresh properties of SCC. In this study, an adaptive neuro-fuzzy inference system (ANFIS) algorithm is developed to predict the superplasticizer (SP) demand and select the most significant parameter of the fresh properties of optimum mix design. For this purpose, a comprehensive database consisting of verified test results of SCC incorporating cement replacement powders including pumice, slag, and fly ash (FA) has been employed. In this regard, at first, fresh properties tests including the J-ring, V-funnel, U-box, and different time interval slump values were considered to collect the datasets. At the second stage, five models of ANFIS were adjusted and the most precise method for predicting the SP demand was identified. The correlation coefficient (R^2^), Pearson’s correlation coefficient (r), Nash–Sutcliffe efficiency (NSE), root mean square error (RMSE), mean absolute error (MAE), and Wilmot’s index of agreement (WI) were used as the measures of precision. Later, the most effective parameters on the prediction of SP demand were evaluated by the developed ANFIS. Based on the analytical results, the employed algorithm was successfully able to predict the SP demand of SCC with high accuracy. Finally, it was deduced that the V-funnel test is the most reliable method for estimating the SP demand value and a significant parameter for SCC mix design as it led to the lowest training root mean square error (RMSE) compared to other non-destructive testing methods.

## 1. Introduction

SCC is a type of concrete that requires a higher dosage of cement and fine aggregate as well as lower coarse aggregate content in comparison with normal concrete [1]. This material provides a high level of workability for structural applications and is a good option for enormous cast volume. On the other hand, high cement content is one of the harmful issues of SCC for the environment. Segregation is another typical problem of employing SCC that is highly sensitive to the water to cement ratio. Different types of SCCs have been proposed by researchers to address mentioned shortcomings [2]. In order to deal with high cement content, various cement replacement powders including either natural or synthetic powders have been proposed and verified by reliable investigations [3,4,5].

Different types of natural and industrial powders are available, which have several advantages and disadvantages. Synthetic powders including silica fume, slag, and fly ash typically enhance the hardened properties of concrete especially the compressive strength, but as long as they are industrial products, their environmental problems encourage researchers to find environmentally friendly alternatives for them. Many natural cementitious materials have been proposed in the past decades such as rice husk ash (RHA), perlite, zeolite, limestone, and pumice. These powders did not present the same performance, and each one has a unique behavior in concrete [6]. Some studies have worked on the evaluation of the properties of SCC containing fly ash and silica fume and reported that silica fume has a noticeable effect on the hardened properties of SCC, especially when incorporated with 10–25% volume content [7,8,9,10].

Pumice is a kind of volcanic product, which is mostly made of silica and alumina, and its components are similar to bubbles with a large inner surface [11]. The physical and chemical properties of pumice result in great durability and strength. Pumice improves not only concrete durability [12] but also shows excellent resistance against sulfate attacks [13]. Concrete can be produced by pumice with high strength and low weight in comparison with concrete made by cement [14,15]. Using pumice powder and slag as cement replacement has been also investigated in another study [16], where SCC incorporated with pumice showed proper sustainability and mechanical properties. However, slag represented better performance compared to pumice. Moreover, SCC incorporating pumice can keep the slump flow in a suitable range and increase the SP dosage in the mixture. Pumice is able to decrease the possibility of segregation and increase the workability retention of the SCC [17]. On the other hand, slag is a mineral product that is chemically similar to cement [18]. There are several types of slag generally divided into two main categories: (1) crystallized slag and (2) granulated blast furnace slag. Low heat in hydration, proper performance, resistance to sulfate attack and acid, resistance to abrasion and corrosion, and reasonable cost [18] are the benefits of slag. Natural powders typically help to maintain workability in SCC especially with increasing cement replacement content up to 30% of total binder volume [19]. According to Zhao et al. [20], partial replacement of cement with slag and fly ash assists the SCC mixture to remain in target slump value. Besides, it was found that slag can decrease the slump loss rate.

Workability retention and slump durability are the most critical factors of a sustainable SCC [21]. To maintain the slump flow in SCC as long as it takes to cast in situ applications, SP content should be considered as a key parameter in the mix design [22]. SP content also plays an important role in the workability retention of SCC. In fact, SP demand is the required value of SP to maintain both the slump flow and workability in structural applications, which is obtained from experimental investigations. Employing natural cement replacements mainly increase the SP demand compared to control SCC samples [23].

Although in recent years, several experimental studies have been carried out to investigate the properties of concrete products incorporating cementitious materials, artificial intelligence (AI) as a human intelligence-based approach can be utilized as assistance for numerical and experimental approaches [24,25,26,27]. The advantage of AI models in many studies has been proven due to providing more reliable results compared to other methods.

In a research study conducted by Uysal and Tanyildizi [28], an artificial neural network (ANN) model was utilized to estimate the loss in compressive strength of the SCCs containing polypropylene (PP) fiber and different types of mineral additives. Promising results were obtained using the ANN model as a reliable alternative instead of experimental methods. Similarly, Asteris et al. [29] proposed an ANN model based on experimental data to estimate the mechanical characteristics of the SCC. The comparative results of this study proved the valuable and reliable use of neural networks in predicting the mechanical properties of SCCs. Golafshani et al. [30] applied the grey wolf optimizer (GWO) in the training phase of ANN and ANFIS models to develop hybridized algorithms for predicting the compressive strength of normal and high-performance concrete. The findings showed improvement in the training phases and generalization capabilities of the proposed models using GWO. Vakhshouri and Nejadi deployed ANFIS models to predict the compressive strength of SCC. They assigned the compressive strength as the output, and slump flow and mixture proportions were considered as inputs. It was reported that the most accurate prediction is obtained for compressive strength when the model includes all input data [31].

In addition, several attempts were carried out to predict the mechanical properties of SCC or determine optimum values of the related parameters to achieve the desired compressive strength. In this regard, a research study conducted by Douma et al. [32] showed correct estimation of fresh SCC properties using the ANN model. Similarly, the research study conducted by Elemam et al. [33] demonstrated the applicability of the ANN model in estimating the fresh and hardened properties of SCC. In another effort by Azimi-Pour et al. [34], support vector machines (SVMs) were used to model the fresh properties of fly ash-based SCC by minimizing the experimental tests.

As mentioned earlier, many papers investigated the fresh properties of SCC incorporating cement replacement materials. Partial replacement of cement in SCC with other materials may lead to changes in the fresh properties. Although these changes can be observed and calculated based on experimental tests, identifying the most influential parameter might not be a straightforward task. To address this problem, the use of artificial intelligence (AI) tools could be helpful. The adaptive neuro-fuzzy inference system (ANFIS) is a form of neural network that can learn and adapt automatically [35]. ANFIS, in contrast to most analytical procedures, does not require the system parameters to be known, and its simpler solutions can be adopted for multivariable problems [36,37,38,39].

### Aim of the Study

According to the literature, the SP demand as one of the controversial parameters of mix design of SCC has not been investigated as much as fresh and hardened properties. For example, Feng et al. [40] examined the SP demand of SCC using hybrid intelligent algorithms and obtained promising results. Additionally, as discussed comprehensively, several studies were performed to predict the different characteristics of concrete using AI models. However, there is no study regarding the most influential parameters on the SP demand. Therefore, this study aims to investigate, estimate, and determine the SP demand and its most effective factors using an AI technique. For this purpose, an ANFIS algorithm is deployed to predict the SP demand of SCC incorporating pumice, slag, and fly ash powders as partial replacements. In addition, several ANFIS models, including 5 models with separate inputs and 21 models with a couple of parameters were trained using experimental data [16]. Finally, the effect of input parameters, i.e., contents of slag, silica fume, pumice, fly ash, cement, and coarse and fine aggregates were investigated.

## 2. Experimental Method

### 2.1. Materials

**Figure 1:**
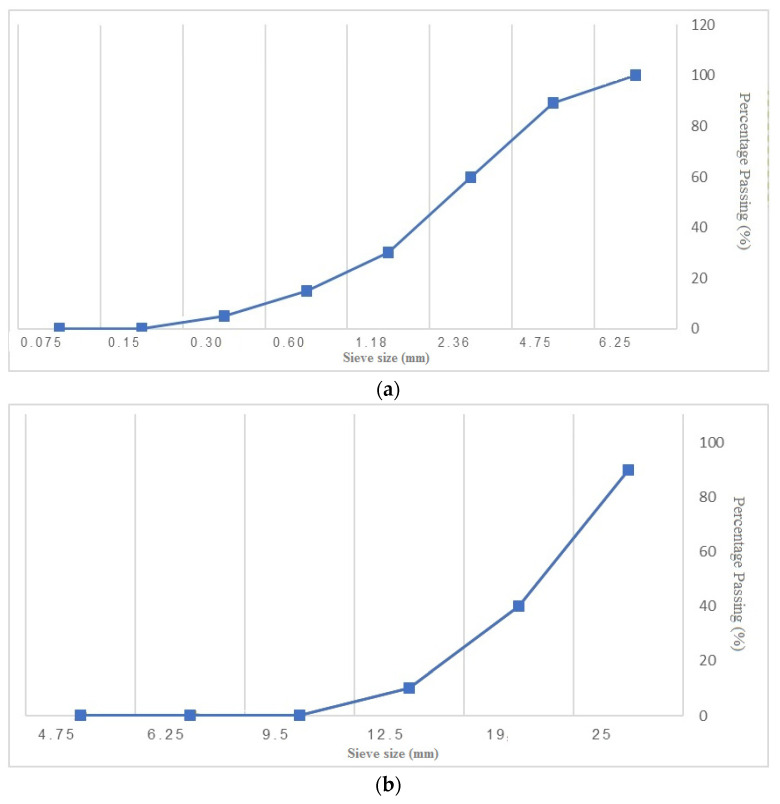
Sieving diagrams of (**a**) fine aggregates and (**b**) coarse aggregates.

The verified experimental results from the literature [16] for a commercially available ASTM type II Portland cement with a specific density of 3160 kg/m^3^ and a fineness of 290 m^2^/kg containing the volcanic pumice were employed. Coarse aggregates were mixed with a maximum size of 19 mm and a density of 2.5 kg/cm^3^, and fine aggregates with a blain of 3.6 m^2^/kg, specific density of 2.7 g/cm^3^, and water absorption of 2.95% were considered. The quality of water can affect the mechanical properties of concrete according to previous studies [41,42,43]. However, in this research, tap/drinking water was employed for sample preparations. The carboxylate-based superplasticizer (SP) with a density of 1.07 g/cm^3^ was applied to obtain a desirable efficiency and regulate the slump loss. As mentioned earlier, pumice, fly ash, slag, and silica fume are the cement alternatives with different replacement percentages, which are used with binary and ternary mixtures. Table 1 reveals the specific density and chemical components of the cement. Figure 1 indicates the sieving analysis of fine and coarse aggregates based on the percentage of passing from the standard sieve in mm.

### 2.2. Mixture

According to [16,40], the selected SCC specimens included fly ash, pumice, and slag as binaries samples with replacement percentages of 10%, 20%, 30%, 40%, and 50% and a water to cement ratio of 38%. In the second series of samples, ternary mixtures of pumice and silica fume with the same water to cement ratio have been employed. In all designs, the cementitious material content is 500 kg/m^2^. In addition, the replacement percentage of each design is shown by its name. Based on the ASTM provisions for concrete production [44], the dry materials are blended firstly, and then SP and water are added. Regarding the EFNARC [45] guidance for fresh properties tests, the mixing process took about 10 min, and after the first 3 min, the concrete rested for 4 min and therefore, SCC was mixed in the machine in 3 min. Finally, the slump flow test was started after 10 min, and the process of slump test was continuously performed at 20, 30, and 40 min.

### 2.3. Test Procedure

Based on ASTM C1611 guidelines [46], the slump test was carried out to identify the workability of the specific samples at different time intervals. The standard slump value is measured after the funnel removal and waiting for the settling of the paste.

## 3. Test Results

### Fresh Properties

**Figure 2:**
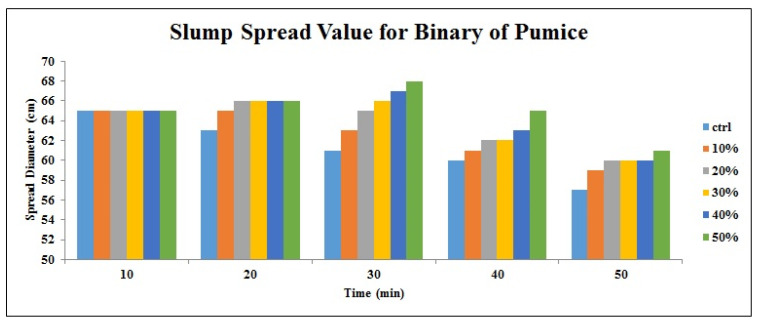
Slump value for pumice powder samples throughout the first 50 min.

**Figure 3:**
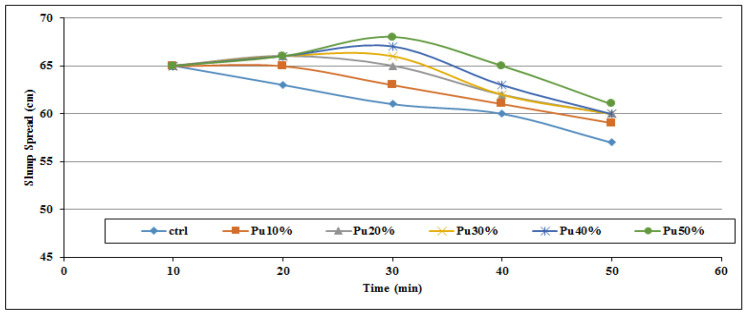
Slump loss diagram for pumice powder samples throughout the first 50 min.

**Figure 4:**
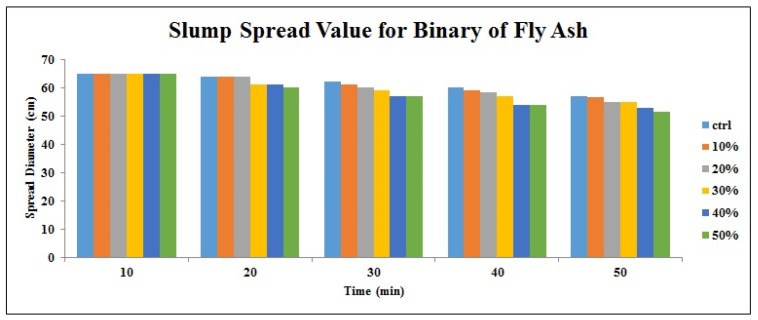
Slump value for FA powder samples throughout the first 50 min.

**Figure 5:**
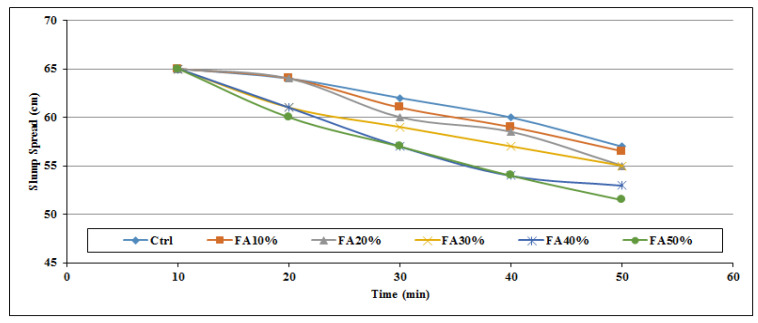
Slump loss diagram for FA powder samples throughout the first 50 min.

**Figure 6:**
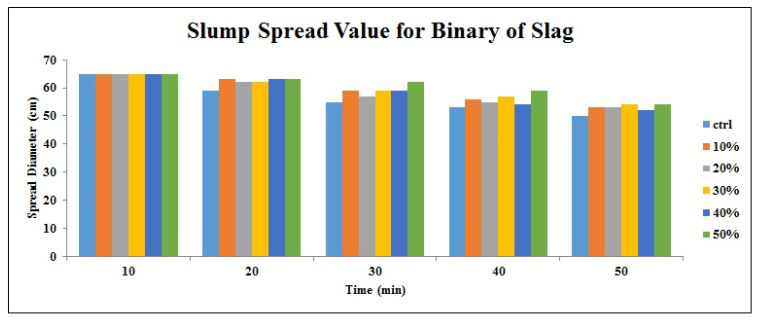
Slump value for slag powder samples throughout the first 50 min.

**Figure 7:**
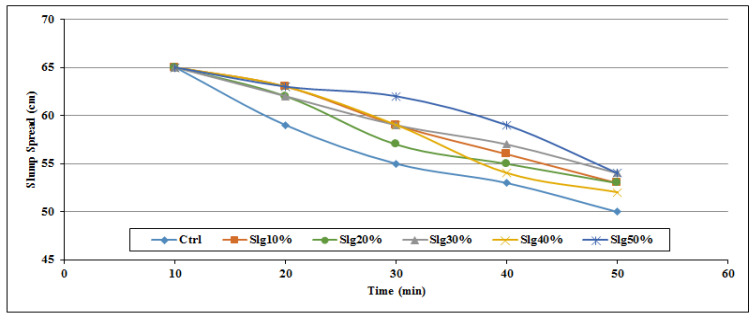
Slump loss diagram for slag powder samples throughout the first 50 min.

Figure 2, Figure 3, Figure 4, Figure 5, Figure 6 and Figure 7 demonstrate a cumulative diagram of slump value between 10 and 50 min for all binaries. According to these figures, each specimen has the same slump spread (±5 cm) compared to the control (ctrl) sample. In Figure 2, pumice has increased the slump flow by 20% replacement, especially in the first 30 min. On the other hand, other replacement dosages have not increased the flow-ability of the concrete noticeably. Even in higher dosages, slump flow experiences a significant loss at 50 min. The slump loss trend of each pumice sample is indicated in Figure 3. According to Figure 4, FA replacement did not increase the slump flow in comparison with control samples; however, FA specimens retain the slump flow along the 50 min on an acceptable range. Figure 5 shows a diagram of slump loss curves for FA specimens, which certify the information in Figure 4. Figure 6 illustrates the slag addition influence on slump loss pattern, where samples in the first 10 min revealed appropriate slump value and could keep the slump in an allowable range until 50 min after initial mixing. Figure 7 shows a diagram for slump values of slag samples.

**Figure 8:**
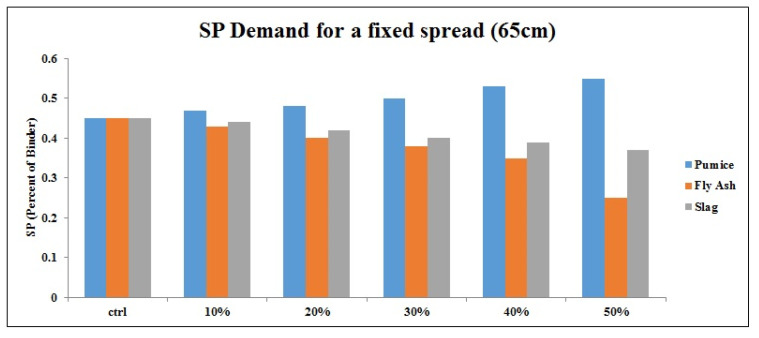
SP consumption of each replacement based on replacement percentage for a fixed slump value.

Since the microparticles of slag absorbed a small amount of water at the beginning of the production process, the whole slag binaries became sensitive to SP dosage. Therefore, even a small dosage of SP over than standard value can lead to concrete segregation. On the other hand, by comparing other results, it seems that slag shows better performance than the other two powders due to the reasonable SP consumption and the slump loss. Due to the smooth geometry of fly ash particles, the FA binaries have the minimum SP demand and high slump loss. This specific shape even helps the samples to show better fluidity [47]. Moreover, according to the chemical properties of FA, binaries of FA require lower curing time that leads to faster slump loss. Based on the test results, pumice samples indicated higher SP demand to achieve a specific slump of 65 ± 2. Figure 8 reveals the cumulative chart of SP consumption of each replacement powder according to the mix proportion for 65 cm as the fixed slump spread. Moreover, in the 10th minute, the SP amount to reach the slump of 65 ± 2 is obtained. In general, it was found that as the replacement percentage increases, the need for SP decreases.

**Figure 9:**
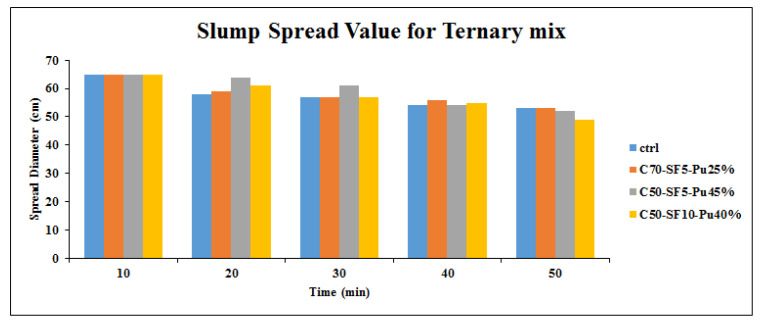
Slump flow value for ternary designs in the first 50 min.

**Figure 10:**
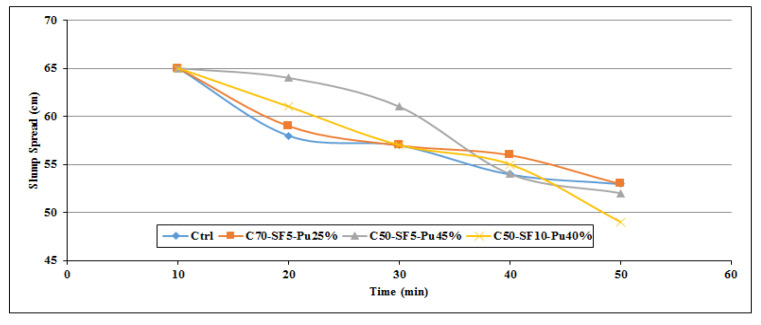
Slump loss diagram for ternary blends in the first 50 min.

Ternary mixes (Figure 9) followed the same pattern as binary samples in slump loss which were indicated in Figure 6. In Figure 9, the slump value has been presented for the first 50 min, where C50-SF5-Pu45% revealed the best performance in retaining the workability and the minimum slump loss. In Figure 10, the slump loss is shown by different curves for ternary mixes. The C50-SF5-Pu45% sample is the optimum mix design based on slump retaining and the workability of SCC.

**Figure 11:**
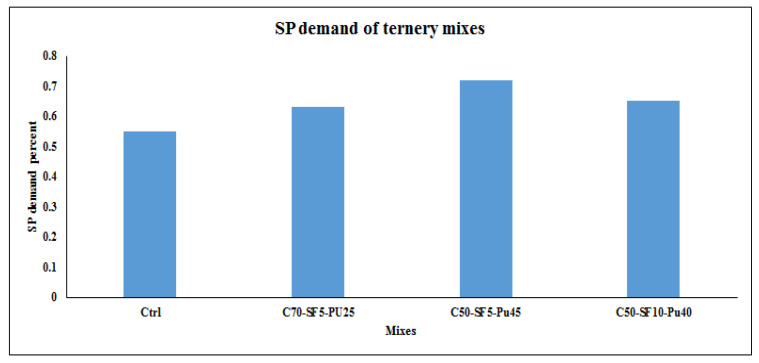
SP consumption of ternary mixes for the fixed 65 cm slump.

Figure 11 indicates the SP demand chart for ternary mixes based on the fixed slump of 65 cm, which reveals that the C50-SF5-Pu45% model has the most demand of SP dosage among other samples.

According to Figure 8 and Figure 11, compared to other powders, by adding more pumice to the admixture, more SP demand is required. Besides, the additional volume of pumice (more than 30%) leads to higher slump loss. In the ternary samples, silica fume did not change the SP demand or the initial slump but had some effects on the slump loss. As a result, in an analogy between ternary mixes of pumice and silica fume, a mixture containing pumice with a higher percentage of replacement requires more SP. Generally, the SP demand of samples had the opposite trend with slump loss, where samples with low SP demand showed significant slump loss. Based on the results, 30% and 45% are optimum replacement percentages for pumice in binary and ternary designs, respectively, while this value is 50% for FA and slag powders.

Table 2 shows the V-funnel results for each sample based on the time taken for admixtures to evacuate the funnel per second. Generally, the V-funnel results indicate the fluidity of concrete by measuring the time taken of concrete to flow from the funnel after 10 s and 5 min of preparing concrete; however, once segregation happens in concrete, the flow time of concrete increases significantly [45].

The U-box test results are revealed in Table 3, which are based on measuring concrete heights in the separate sections of the U-box after pulling up the separator plate by calculating the difference (H2–H1).

In Table 4, J-ring test reports for each sample are indicated. The J-ring flow test measures the diameter of flow and the difference between concrete height inside and outside the J-ring (H2–H1) [16].

Finally, the ternary results for J-ring, V-funnel, and U-box tests are presented in Table 5.

## 4. ANFIS Methodology

**Figure 12:**
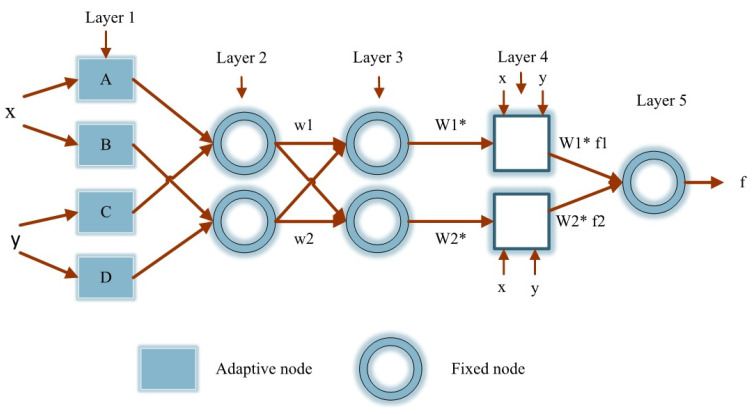
ANFIS layers [49].

ANFIS is a fuzzy inference system [48] that is developed in an adaptive network framework. The ANFIS network is made up of five levels, as shown in Figure 12 [49]. The fuzzy inference system is generally located at the core of the ANFIS network. The first layer takes inputs (x and y in Figure 12) and employs membership functions to transform them to fuzzy values [50,51,52,53]. The Takagi–Sugeno style IF-THEN rules are presented as follows:

Rule 1: if x is $A_{1}$ and y is $B_{1}$, then $f_{1}=p_{1}x+q_{1}y+r_{1}$,

Rule 2: if x is $A_{2}$ and y is $B_{2}$, then $f_{2}=p_{2}x+q_{2}y+r_{2}$,

Every node in this layer (the first) is chosen as an adaptive node with a node function:

(1) $$O_{i}^{1}=\mu A_{i}\left(x\right)$$

where $A_{i}$ is a linguistic label and $O_{i}^{1}$ is the membership function of $A_{i}$. The bell-shaped membership function is usually selected as it has the highest capacity for the regression of nonlinear data [54]. Bell-shaped membership function with the maximum value of 1 and minimum value of 0 is defined as follows:

(2) $$\mu \left(x\right)=bell\left(x;a_{i},b_{i},c_{i}\right)=\frac{1}{1+{\left[{\left(\frac{x-c_{i}}{a_{i}}\right)}^{2}\right]}^{b_{i}}}$$

where $\left\{a_{i},\;b_{i},c_{i},d_{i}\right\}$ are the parameters set and x is the input. Premise is defined as the parameter of this layer. The second layer multiplies the input signals before sending the result to the next layer. Consider the following example:

(3) $$w_{i}=\mu A_{i}\left(x\right)\times \mu B_{i}\left(y\right),\;i=1,2\ldots$$

The firing strength of a rule may be seen in each node’s output. The rule layer is the third and final layer. The ratio of the $i^{th}$ node’s rule firing strength to that of the other nodes is computed in this layer as follows:

(4) $$w_{i}^{*}=\frac{w_{i}}{w_{1}+w_{2}}\;i=1,2\ldots$$

where $w_{i}^{*}$ is referred to as normalized firing strength. The defuzzification layer is the fourth layer, in which each node has a node function, as presented below:

(5) $$O_{i}^{4}=w_{i}^{*}f_{i}=w_{i}^{*}\left(p_{i}x+q_{i}y+r_{i}\right)$$

where $\;w_{i}^{*}$ is the third output layer and $\left\{p_{i},q_{i},r_{i}\right\}$ are defined as the consequent parameters. The output layer is the fifth layer. The overall output is calculated in this layer by summing all of the input signals. That is to imply:

(6) $$O_{1}^{5}=f=\underset{i}{\sum }w_{i}^{*}f_{i}$$

During this process, a threshold between the real value and the output is defined. Then, using the least-squares approach, the consequent parameters are calculated, and an error for each data set is determined. If this value is greater than the specified threshold, the premise parameters will be updated using the gradient descent method. This procedure is repeated until the error reaches below the threshold. The utilized approach in this procedure is known as a hybrid algorithm since two algorithms (i.e., least-squares and gradient descent algorithm) generate the parameters concurrently.

### 4.1. Precision Criteria

In this study, several performance metrics were utilized to assess the precision of the proposed models. In this regard, correlation coefficient (R^2^), Nash–Sutcliffe efficiency (NSE), Pearson’s correlation coefficient (r), Wilmot’s index of agreement (WI), root mean square error (RMSE), and mean absolute error (MAE) were considered as follows:

(7) $$R^{2}=\frac{\sum_{i=1}^{M}\left(O_{i}-\bar{O_{i}}\right).\left(P_{i}-\bar{P_{i}}\right)}{\sqrt{\sum_{i=1}^{M}{\left(O_{i}-\bar{O_{i}}\right)}^{2}\sum_{i=1}^{M}{\left(P_{i}-\bar{P_{i}}\right)}^{2}}\;}\;\left[\mathrm{Range}\;=\left(0–1\right);\;\mathrm{superior}\;\mathrm{value}\;=1\right]$$

(8) $$\mathrm{NSE}=1-\frac{\sum_{i=1}^{M}{\left(P_{i}-O_{i}\right)}^{2}}{\sum_{i=1}^{M}{\left(O_{i}-\bar{O_{i}}\right)}^{2}}\left[\mathrm{Range}\;=\left(-\infty ,\;1\right);\;\mathrm{superior}\;\mathrm{value}\;=1\right]$$

(9) $$\mathrm{RMSE}=\sqrt{\frac{1}{M}\overset{M}{\underset{i=1}{\sum }}{\left(P_{i}-O_{i}\right)}^{2}}\;\left[\mathrm{Range}\;=\left(0,+\infty \right);\;\mathrm{superior}\;\mathrm{value}\;=0\right]$$

(10) $$\mathrm{MAE}=\frac{\sum_{i=1}^{M}\left|P_{i}-O_{i}\right|}{N}\;\left[\mathrm{Range}\;=\left(0,+\infty \right);\;\mathrm{superior}\;\mathrm{value}\;=0\right]$$

(11) $$\mathrm{WI}=1-\frac{\sum_{i=1}^{M}{\left(O_{i}-P_{i}\right)}^{2}}{\sum_{i=1}^{M}{\left(\left|P_{i}-\bar{O_{i}}\right|+\left|O_{i}-\bar{O_{i}}\right|\right)}^{2}}\left[\mathrm{Range}\;=\left(0,1\right);\;\mathrm{outstanding}\;\mathrm{value}\;=1\right]$$

(12) $$r=\frac{M(\sum_{i=1}^{M}O_{i}.P_{i})-(\sum_{i=1}^{M}P_{i}).(\sum_{i=1}^{M}O_{i})}{\sqrt{\left(M\sum_{i=1}^{M}O_{i}^{2}-{\left(\sum_{i=1}^{M}\bar{O_{i}}\right)}^{2}).(M\sum_{i=1}^{M}P_{i}^{2}-{\left(\sum_{i=1}^{M}\bar{P_{i}}\right)}^{2}\right)}\;}\;\;\left[\mathrm{Range}\;=\left(0–1\right);\;\mathrm{superior}\;\mathrm{value}\;=1\right]$$

where $O_{i}$ and $P_{i}$ are measured and estimated values, respectively. Additionally, $\bar{O_{i}}$ and $\bar{P_{i}}$ are mean of the measured and estimated values, respectively.

Nash–Sutcliffe (NS) efficiency is a normalized statistic that determines the relative amount of residual variance compared to the variance of the calculation (Nash and Sutcliffe [55]). The Nash–Sutcliffe performance shows how well the observed data graph versus the simulated one corresponds to a 1:1 line. NS = 1 corresponds to the model of full compliance with the observed data. NS = 0 indicates that the model predictions are as accurate as the average of the observed data. 0 < NS < ∞ indicates that the observed average is a better prediction of the model. Mean absolute error (MAE) and mean square error (RMSE) are two of the most common criteria used to measure the accuracy of continuous variables. MAE measures the average size of errors in a set of predictions regardless of their direction. This average test is the absolute difference between prediction and actual observation that all individual differences have equal weight. RMSE is a quadratic scoring rule that also measures the average error rate. This square root is the average square difference between prediction and actual observation [56]. From an interpretation point of view, MAE is the winner. RMSE does not describe moderate error alone and has other implications that are more difficult to understand. On the other hand, one of the distinct advantages of RMSE over MAE is that RMSE avoids the use of absolute values, which is undesirable in many mathematical calculations [57]. Correlation coefficients typically measure the scattering of data against the standard deviation and draw a virtual envelope line across the data in a Cartesian system. Based on the quality of difference between vertical axis number and its corresponding horizontal axis number, the correlation value varies between zero and one while the one is the best correlation coefficient and zero means no relation between numbers [58,59].

### 4.2. Dataset Arrangement

The used data in this investigation was obtained from the conducted tests on the specimens [16]. Totally, a database containing 340 datasets was collected. The results of the J-ring, U-box, and V-funnel tests and slump values in the 3rd and 50th minutes were considered as the inputs of the models, and the SP demand was set as the output. Table 6 shows some details of the dataset.

### 4.3. Development of Models

In order to identify the most effective parameters on the SP demand, 5 main models were established, while 21 dataset models with multi-parameters and 340 samples were developed and examined. After training different models, the results were compared, and finally, five models were derived to predict the SP demand. The mentioned models include inputs according to Table 6, where the first model comprised input 1, the second model included both input 1 and input 2, and this sequence continues until model 5. This arrangement was derived from test and trial procedures based on the quality of precision coefficient from each model with a specific arrangement. Table 7 shows the input parameters based on the quality of arrangement in each model. According to Table 7, model 5 has all five parameters as inputs while model 1 includes only one input (j-ring).

Since the algorithm has to be developed by collected data, model 3 was selected randomly to be adjusted based on the best possible results. After the adjustment, the ANFIS algorithm was developed according to the new parameters. Considering the number of data and avoiding overfitting, 75% of the inputs were randomly devoted to the training phase of the models, and the remaining 25% were assigned to the testing phase. All the codes were developed in the MATLAB environment, and available functions of the MATLAB software (R2019a) were used in the developing process.

### 4.4. Results and Discussion

**Figure 13:**
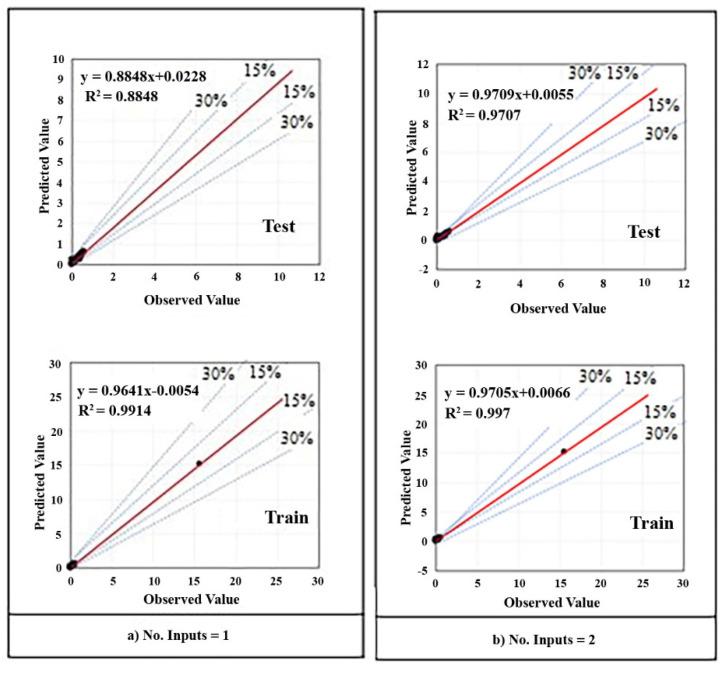

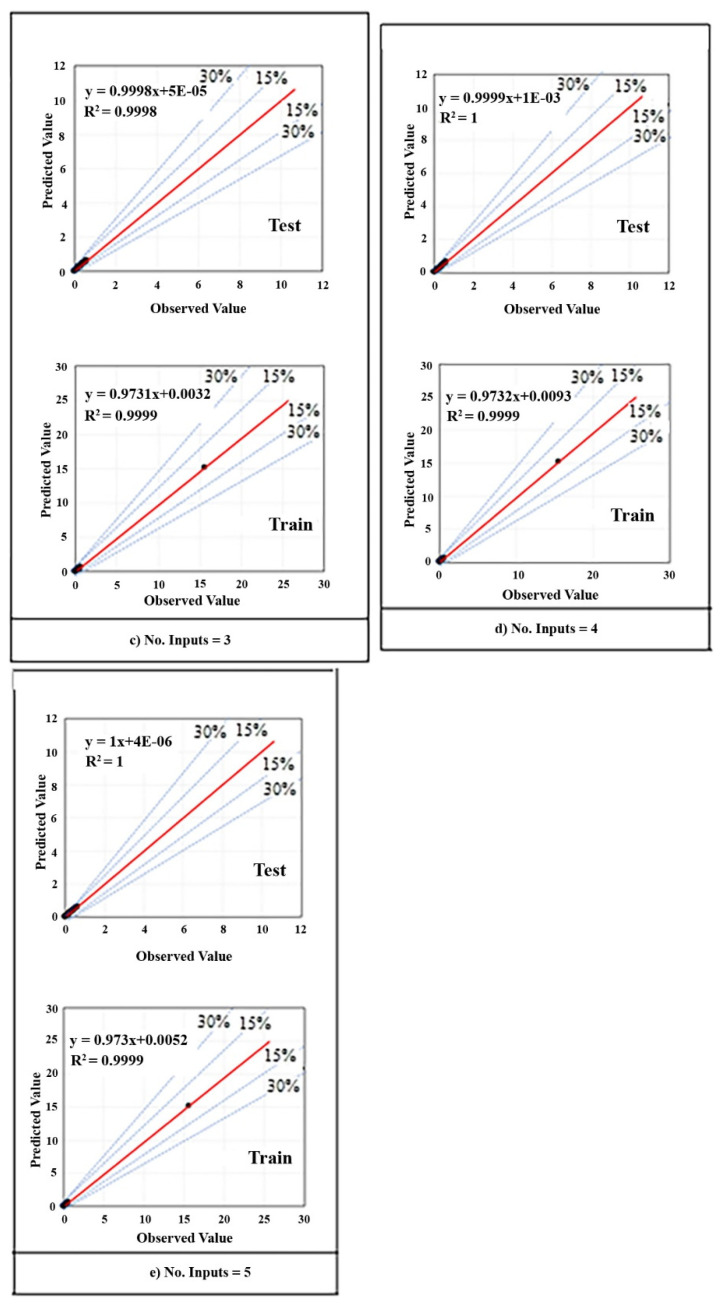
ANFIS regression diagrams: (**a**) single input; (**b**) two inputs; (**c**) three inputs; (**d**) four inputs; (**e**) five inputs.

The impact of each input variable on the output variable can be observed by the RMSE value. The model with the lowest value of RMSE in the training phase demonstrates a better ability to solely predict the output. Each of the ANFIS models was run three times and the mean value of RMSEs in the training and testing phases were recorded. Table 8 reveals the calculated accuracy criteria for the performance of the implemented models based on advanced input parameters. As can be seen in this table, model 5 is the most effective parameter on the output, which has the lowest value of RMSE in the training phase. In other words, the V-funnel test is the best indicator in the prediction of SP demand. Figure 13 indicates the regression scatter diagrams of prediction results for each model, and the relation between observed (experimental) value and predicted value is written as an equation in each diagram. Besides, diagrams are separated into two single parts including train chart and test chart and the dispersion percent is clarified by guidance line around the envelope line (red line). Although all of the charts represent a suitable estimation, the best chart is obviously Figure 13e which has been also presented in Figure 17 separately.

As shown in Table 8 and Figure 13, the best performance parameters in the testing phase for ANFIS are RMSE = 0.001, r = 1.000, R^2^ = 1.000, NSE = 1.000, MAE = 0, and WI = 1.000. Models 4 and 5 represented the best results, and in both models, the V-funnel data has been added to other inputs. The best result for RMSE is the lowest value, and for r, the best positive correlation coefficient is 1, which means that the numbers closer to 1 are considered better results. Furthermore, the smaller values for NSE and MAE, and greater values for WI indicate better performance. Additionally, it can be observed that after model 5, model 4 has shown the lowest training MAE. Therefore, the slump value is the second most effective parameter in the SP demand prediction. The order and the efficiency of other inputs on the SP demand value can also be observed in Figure 13.

**Figure 14:**
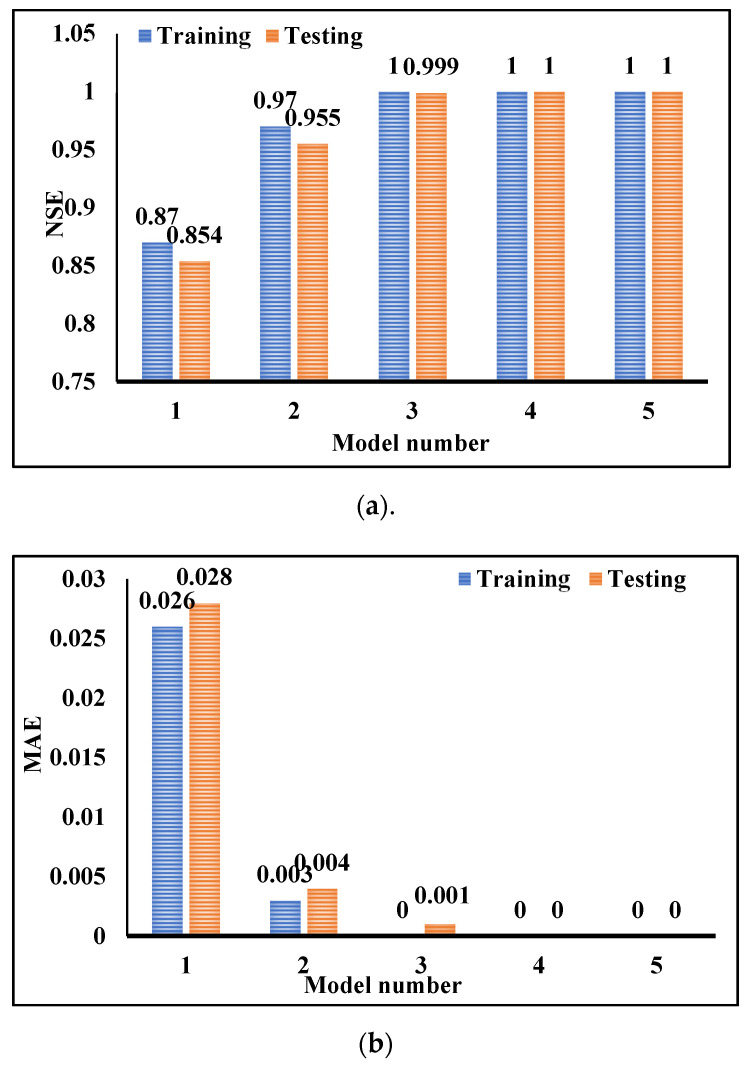
Evaluation values in the training and testing phases: (**a**) NSE; (**b**) MAE.

Figure 14 indicates the clustered charts of some precision parameters based on training and test results. In this figure, the MAE and NSE values of the testing and training phases for each model are displayed. By comparing their values in both phases, it can be concluded that the developed models have revealed an appropriate performance and overfitting has been avoided. All three parameters demonstrate that models 4 and 5 performed better than other models.

**Figure 15:**
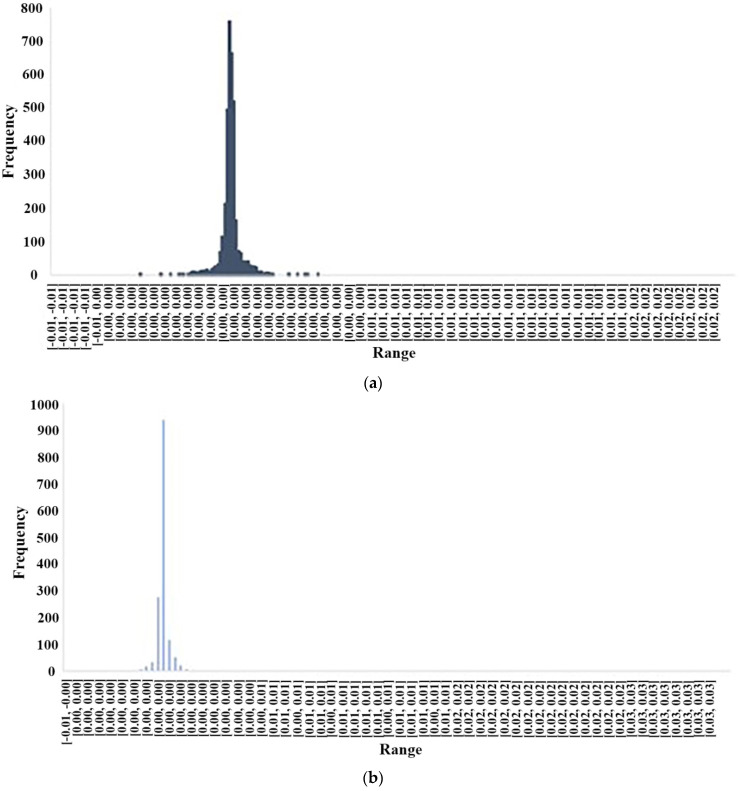
ANFIS error histograms for the best model: (**a**) train phase and (**b**) test phase.

Figure 15 depicts the error histogram for model 5. This figure could prove the accuracy of the model in each training and testing phase again. It was found that ANFIS had an accurate prediction since both the training and testing phases have the same pattern (convergence) in the same area of the samples, and also the difference between predicted and measured values is not noticeable.

**Figure 16:**
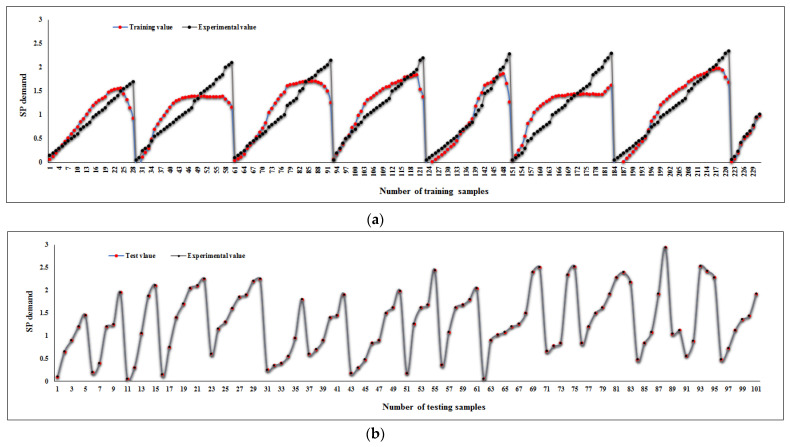
Tolerance diagram of predicted and measured values for (**a**) training phase and (**b**) testing phase.

Figure 16 indicates a tolerance diagram of predicted and measured values for both testing and training phases, which contains a comparison of outputs and targets for the selected ANFIS model. Figure 16b certifies the reliable prediction of model 5 and the noticeable accuracy of the ANFIS.

**Figure 17:**
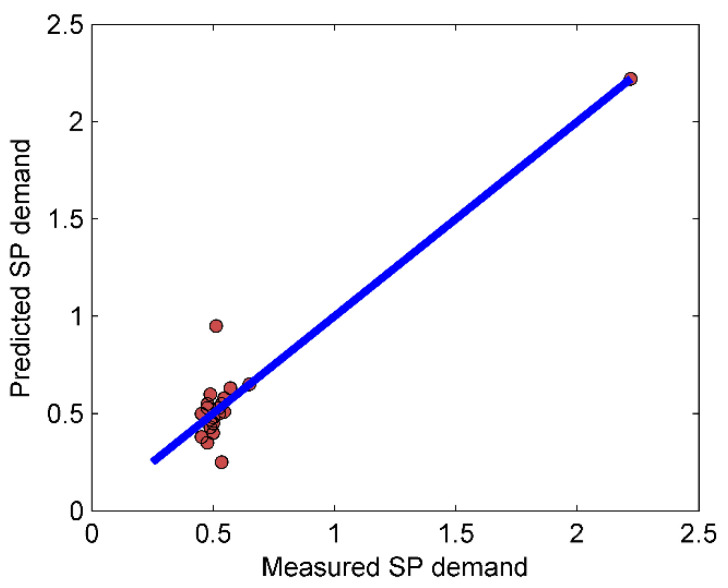
Scatter plot of the SP demand prediction.

Figure 17 shows the scatter plot of the predicted results for the SP demand of the model, which obtained the highest rate among other inputs. In this diagram, the values of RMSE, r, and R^2^ are equal to 0.001, 1.000, and 0.999, respectively. These values illustrate that developing an ANFIS model can be an efficient approach in SP demand estimation.

## 5. Conclusions

The prediction of superplasticizer (SP) demand is complicated due to the many factors involved in the estimation problem. Hence, in this paper, a soft computing methodology was employed, which is more accurate and reliable than other numerical and experimental methods. For the first time, an AI technique was used to select the most influential parameter in the SCC design. In order to achieve a reliable database, verified data from an experimental program was used to investigate the possible incorporation of pumice, slag, and fly ash powders as cement replacements in binary and ternary mixtures. To this end, different approaches, including the results of J-ring, U-box, V-funnel, and slump tests were considered to predict the SP demand value. After comparing the different types of inputs, five key characteristics of the SCC were selected as the most influential inputs, which were J-ring, U-box, V-funnel, 3 min slump, and 50 min slump. In continue, five ANFIS models were established and the impact of each model on the SP demand prediction was evaluated. In general, it was found that the ANFIS can accurately predict the results of the experiments. In addition, the ANFIS parameters were kept constant (clusters = 10, train samples = 75%) to compare the five ANFIS models. A summary of the obtained results are presented as follows:

- Pumice showed the highest effect on the SP demand of admixture, in 50% replacement binaries, it has increased the SP demand by 27% and 45% compared to slag and FA, respectively. This higher SP dosage could be related to the geometry shape and structure of pumice particles.
- The optimum content based on the results of fresh properties in binary samples was 30% for pumice and 50% for slag and FA.
- According to neural network results, the pumice incorporation has the most effect on the maintenance of the SCC slump to keep the optimal performance. The best prediction model was determined and the most accurate parameters for RMSE, r R^2^, NSE, MAE, and WI were 0.999, 1.000, 1.000, 0.001, 0.000, and 1.000, respectively.
- Finally, among the five ANFIS models, the model corresponding to the V-funnel test led to the best RMSE, MAE, and NSE values. The results indicate that the V-funnel value is the most influential parameter in the SP demand prediction and SSC design.

## Figures and Tables

**Table 1:** Chemical ingredients of binder aggregates [40].

Components (%)	Cement	Slag	Pumice	SF ^1^	FA ^1^
SiO2	22.42	33.1	44.13	86.2	62.8
Al2O3	4.68	13.8	16.71	1.44	45.9
Fe2O3	3.68	3.12	1.72	0.2	0.92
CaO	63.25	40.7	11.09	3.06	2.60
MgO	3.63	8.70	1.95	1.32	1.40
SO3	1.74	0.60	0.39	0.34	0.49
Specific gravity (kg/m^3^)	3160	2850	2850	2350	2200
Blaine (m^2^/kg)	290	445	320	20000	260

^1^ SF: silica fume, FA: fly ash.

**Table 2:** V-funnel test value for each binary samples.

V-Funnel	Ctrl	10%	20%	30%	40%	50%
Pumice	5	5	5	7	8	9
FA	5	5	5	7	7	8
Slag	6	9	10	10	7	7

**Table 3:** U-box test value for each binary samples.

U-Box	Ctrl	10%	20%	30%	40%	50%
Pumice	0.5	1	1	1.5	2	2.5
FA	0.5	1	1	1.5	2	2
Slag	3	8	5	4	3	3

**Table 4:** J-ring test value for each binary samples.

J-Ring	Ctrl	10%	20%	30%	40%	50%
Pumice	1	1	2	2	3	3
FA	1	1	1	2	3	3
Slag	1	1	1	1	1	1

**Table 5:** Non-destructive tests value for each ternary samples.

Ternaries	Ctrl	C70-SF5-Pu25%	C50-SF5-Pu45%	C50-SF10-Pu40%
J-ring	1	1	1	1
U-box	4	6	2	5
V-funnel	5	5	6	5

**Table 6:** Details of the input and output variables.

Inputs and Outputs	Variables	Minimum	Maximum	Mean Value	Standard Deviation
1	j-ring (mm)	0.70	6.15	2.71	1.44
2	u-box (mm)	0.50	25.00	4.12	5.19
3	50 min Slump (mm)	5.00	60.00	8.73	10.58
4	3 min Slump (mm)	41.00	66.00	52.85	6.72
5	V-funnel (s)	43.00	62.00	55.81	4.49
Output	SP Demand (mm)	0.25	2.22	0.61	0.37

**Table 7:** Arrangement of the models.

Parameter	Number of Model				
	1	2	3	4	5
j-ring (mm)	*	*	*	*	*
u-box (mm)		*	*	*	*
50 min Slump (mm)			*	*	*
3-min Slump (mm)				*	*
V-funnel (s)					*

* shows the used parameter in each model.

**Table 8:** Details of the input and output variables.

Input. Parameters	Network Result											
	Training Phase						Testing Phase					
	R^2^	r	NSE	RMSE	MAE	WI	R^2^	r	NSE	RMSE	MAE	WI
j-ring (mm)	0.885	0.941	0.870	0.035	0.026	0.969	0.991	0.935	0.854	0.038	0.028	0.965
u-box (mm)	0.971	0.985	0.970	0.018	0.003	0.993	0.997	0.978	0.955	0.022	0.004	0.989
50 min Slump (mm)	0.999	1.000	1.000	0.002	0.000	1.000	0.999	1.000	0.999	0.003	0.001	1.000
3-min Slump (mm)	1.000	1.000	1.000	0.001	0.000	1.000	**0.999**	**1.000**	**1.000**	**0.001**	**0.000**	**1.000**
V-funnel (s)	**1.000**	**1.000**	**1.000**	**0.000**	**0.000**	**1.000**	**0.999**	**1.000**	**1.000**	**0.001**	**0.000**	**1.000**

## Data Availability

Data sharing is not applicable to this article.
